# A Multi‐Factor Habitat Suitability Model for Asian Elephants in the Greater Mekong Subregion: Effects of Vegetation and Climate

**DOI:** 10.1002/ece3.73793

**Published:** 2026-06-16

**Authors:** Churui Li, Xiaorui Wang, ZhenPing Qiang, Yongjing Tang, Song Yang, Chenglong Luo, Xiaoyun Tu, Shuang Zhang, Yuting Xia, Lanzhong Zhang

**Affiliations:** ^1^ Southwest Forestry University Kunming China; ^2^ Liangshan Academy of Forestry and Grassland Xichang China; ^3^ Southwest Survey and Planning Institute of National Forestry and Grassland Administration, Asian Elephant Research Center of National Forestry and Grassland Administration Kunming China; ^4^ Jiangcheng County Forestry and Grassland Bureau Pu'er China; ^5^ Institute of International Rivers and Eco‐Security Yunnan University Kunming China

**Keywords:** climate change, conservation planning, *Elephas maximus*, food‐plant resources, habitat suitability, species distribution model

## Abstract

The habitat suitability of the endangered Asian elephant ( *Elephas maximus* ) is commonly assessed using climatic and topographic variables, whereas food‐plant resources are rarely incorporated explicitly into regional‐scale species distribution models. Here, we developed a comparative modeling framework to evaluate how the inclusion of food‐plant resource layers modifies Asian elephant habitat suitability predictions under current and future climate scenarios. (1) The model incorporating food‐plant variables produced a more restricted and spatially concentrated pattern of suitable habitat than the model excluding food‐plant variables. (2) This contrast suggests that climate‐dominated models may overestimate usable habitat in areas where key forage resources are limited or absent. (3) In addition, piecewise SEM suggested that the associations among climate, topography, food‐plant resources, and predicted habitat suitability varied along latitudinal gradients. These findings indicate that food‐plant resource layers can refine climate‐based habitat suitability assessments by distinguishing broad climatic suitability from more realistic resource‐based suitability. Conservation planning should therefore integrate food‐plant restoration, habitat connectivity, and climate adaptation to improve habitat management and reduce human‐elephant conflict.

## Graphical abstract

This study assessed the combined effects of climate change and vegetation factors on the habitat suitability of Asian elephants ( *Elephas maximus* ) in the Greater Mekong Subregion. By integrating MaxEnt modeling with structural equation modeling, we identified plant distribution as a key determinant of elephant habitat selection under future climate scenarios. Our findings highlight the importance of vegetation‐focused and latitude‐specific conservation strategies to mitigate the impacts of climate change and human–elephant conflict.

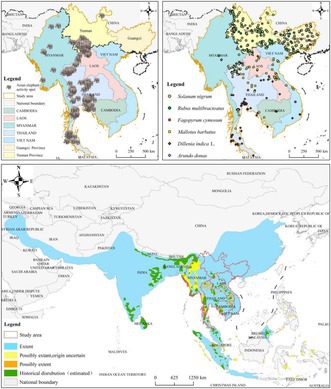

## 1 Introduction

The Asian elephant ( *Elephas maximus* ) is a large herbivore and ecological engineer in tropical and subtropical forest ecosystems, contributing to seed dispersal, vegetation dynamics, and forest regeneration (Chen et al. 2006). Asian elephants face multiple threats, including habitat loss, habitat fragmentation, reduced food availability, and increasing human‐elephant conflict (De et al. 2021). Although forest protection and restoration programs can increase forest cover, they may not always improve the availability of understory forage plants required by Asian elephants (Zhang et al. 2024). In some regions, population recovery under limited habitat availability may further increase pressure on local food‐plant resources (Zhang et al. 2024; Joshi et al. 2025). Under these combined pressures, elephants may increasingly use agricultural landscapes, thereby intensifying human‐elephant conflict (Zhang et al. 2024). These patterns highlight the need for habitat suitability models that consider not only climatic conditions but also the spatial availability of food‐plant resources.

Food availability is a key component of Asian elephant habitat suitability (Sampson et al. 2018; Huang et al. 2019). Because of their large body size and high energetic demands, Asian elephants require abundant and seasonally available forage resources (Clauss et al. 2003; Bao et al. 2021). Therefore, the spatial availability of food plants may help explain why some climatically suitable areas are not equally usable by elephants (Clauss et al. 2003; Liu et al. 2025). Asian elephants consume diverse plant resources, but only a subset of food plants can be represented spatially and incorporated into regional habitat models. Previous diet studies have shown that food‐plant use varies among regions and reflects local floristic composition, seasonal resource availability, and habitat conditions (Jothish 2013; Jiang et al. 2019; Campos‐Arceiz and Blake 2011; Chen et al. 2006; Sekar et al. 2016; Koirala et al. 2016). In addition, elephant habitat use and movement may be linked to the phenology and spatial availability of key forage plants (Zong et al. 2014; Liu et al. 2025; Lin et al. 2008). Therefore, food‐plant predictors should be selected using both dietary evidence and regional occurrence data, and incorporating spatially explicit food‐plant information may improve the ecological realism of Asian elephant habitat suitability models.

**FIGURE 1:**
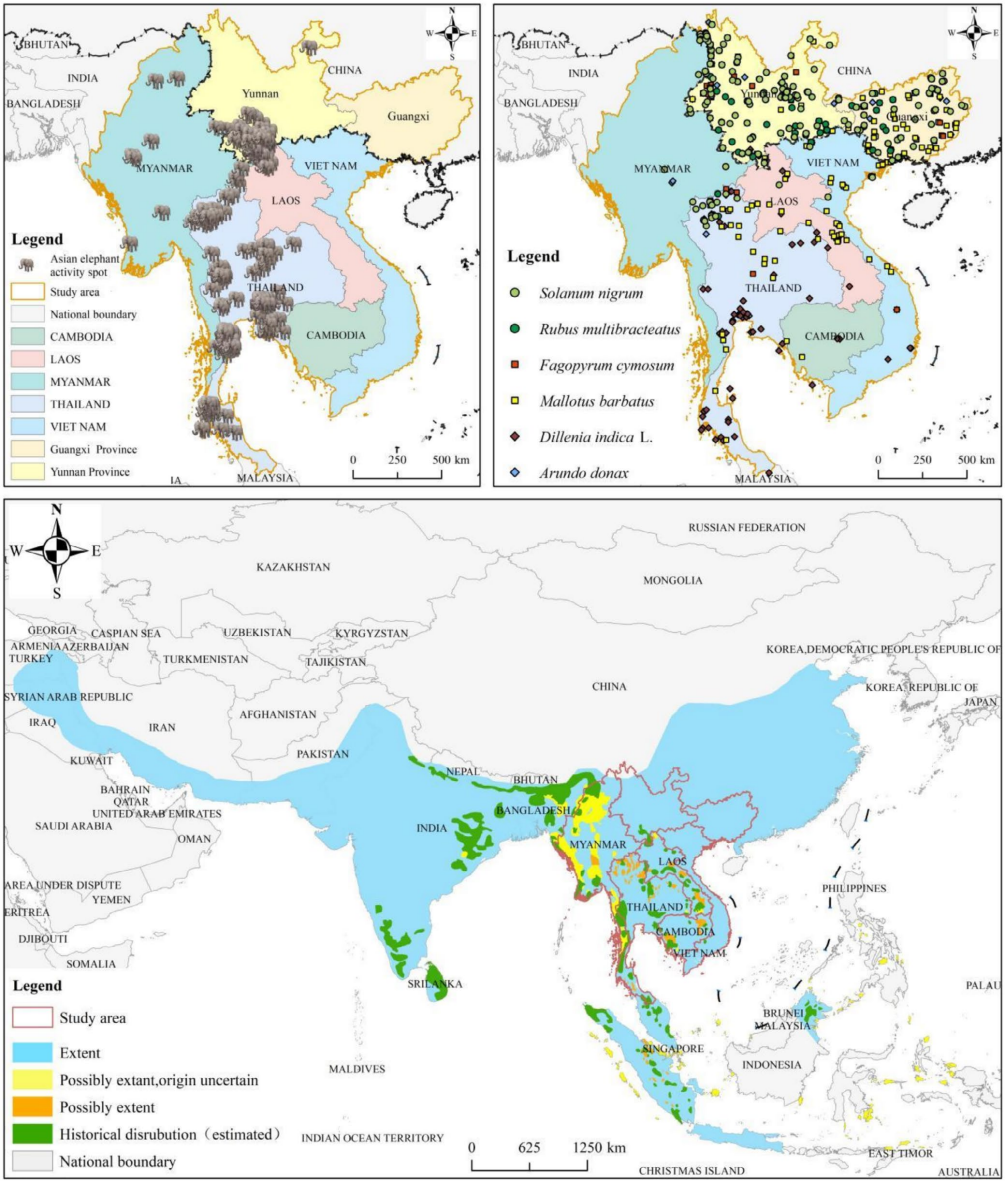
Geographic location of the study area, occurrence records of Asian elephants and selected food plants, and the historical and current distribution of Asian elephants in Asia. The figure shows Asian elephant activity records in the Greater Mekong Subregion, occurrence records of selected food‐plant species, and the historical and current distribution ranges of Asian elephants in Asia. The historical and current distribution map was compiled from Tang et al. (2023), and Zhang et al. (2025).

Although food availability is widely recognized as important for Asian elephants, most regional‐scale habitat suitability models still rely primarily on climatic, topographic, and land‐cover variables. This creates a modeling gap: it remains unclear how explicitly incorporating food‐plant distributions changes habitat suitability predictions and future conservation assessments for Asian elephants. The novelty of this study is therefore not to demonstrate that elephants depend on plants, but to quantify how food‐plant resource layers modify model performance, predicted habitat extent, spatial configuration, ecological niche patterns, and conservation priorities. Specifically, we compared MaxEnt models with and without food‐plant variables under current and future climate scenarios. We evaluated whether food‐plant variables altered model performance, variable contribution, habitat area, and spatial configuration. We further examined ecological niche differences and latitudinal associations among climate, topography, food‐plant resources, and predicted elephant habitat suitability. This comparative framework allows us to distinguish broad climatic suitability from more realistic resource‐based habitat suitability. The results provide spatial guidance for food‐plant restoration, habitat connectivity planning, and human‐elephant conflict mitigation (Figure 1).

## 2 Materials and Methods

### 2.1 Data Collection and Screening

#### 2.1.1 Data Collection

##### 2.1.1.1 Species Occurrence Data

Distribution data for Asian elephants were compiled from published literature and multiple databases, including the Yunnan Pu'er Wildlife Asian Elephant Monitoring Platform and the Global Biodiversity Information Facility (GBIF). An initial set of 6500 occurrence records was collected. To reduce spatial sampling bias, duplicate records and spatially clustered records were removed using the R package spThin, retaining only one record per grid cell (Zhao, Liu, et al. 2025; Li, Li, et al. 2025). This process yielded 780 spatially independent occurrence sites for subsequent analysis. Occurrence records for candidate food‐plant species were compiled from GBIF, published diet studies, regional floristic records, and expert consultation. Candidate food‐plant species were selected because they had been reported as forage resources for Asian elephants and had georeferenced occurrence records within the Greater Mekong Subregion. Species names were standardized using accepted botanical nomenclature before analysis, and records with missing coordinates, duplicated coordinates, or obviously erroneous coordinates were removed.

##### 2.1.1.2 Environmental Variables

Topographic data were obtained from the United States Geological Survey (USGS) at a 30 m resolution. Because terrain is assumed to change negligibly over the modeled timeframes, topography was held constant for all future projections. Climatic variables were obtained from the WorldClim database 2.1 at a 1‐km resolution. Future climate projections for the 2060s, 2080s, and 2100 s were derived from the National Climate Center (Beijing) medium‐resolution CMIP6 models. All data layers were resampled to a consistent 1 km resolution, and three Shared Socioeconomic Pathways (SSPs) were selected for analysis.

##### 2.1.1.3 Food‐Plant Variables Data

We initially selected 15 candidate food‐plant species consumed by Asian elephants based on published dietary records, regional foraging studies, and expert knowledge. These species were selected because they were documented as natural food plants consumed by Asian elephants rather than cultivated crops. Previous studies have shown that Asian elephants are broad‐spectrum herbivores and consume a wide range of natural plant species, including grasses, bamboo, shrubs, fruits, leaves, bark, roots, and rhizomes (Shoshani and Eisenberg 1982; Campos‐Arceiz et al. 2008; Chen et al. 2006; Sampson et al. 2018; Huang et al. 2019). Regional diet studies further indicate that elephant food‐plant use varies with local floristic composition and seasonal resource availability (Jothish 2013; Jiang et al. 2019; Campos‐Arceiz and Blake 2011; Zong et al. 2014; Lin et al. 2008; Table 1). Candidate species were retained if they met three criteria: (1) documented evidence of consumption by Asian elephants in published dietary or regional ecological studies; (2) natural occurrence within the Greater Mekong Subregion; and (3) availability of sufficient georeferenced occurrence records for distribution modeling. The selected species represented different food‐resource types, including herbs, graminoids, bamboo, shrubs, and fruit‐bearing trees. They included plant taxa associated with high‐energy or seasonally important forage resources, such as *Fagopyrum cymosum*, *Solanum nigrum* , *Dillenia indica* L., *Dendrocalamus hamiltonii*, *Bombax ceiba* , *Cratoxylum cochinchinense*, and *Aporosa yunnanensis* (Chen et al. 2006; Sekar et al. 2016; Koirala et al. 2016; Liu et al. 2025). The potential distribution of each candidate food‐plant species was modeled using MaxEnt. Each candidate food‐plant model was evaluated using AUC, ecological relevance, and redundancy with other food‐plant predictors. AUC was used to assess model discrimination ability, but the final selection was not based on AUC alone. Species with poor predictive performance, insufficient occurrence records, limited ecological relevance to elephant foraging, or high redundancy with other food‐plant layers were excluded from the final elephant habitat model (Merow et al. 2017; Li, Wang, et al. 2025). Six food‐plant variables were retained for the final comparative analysis: *Fagopyrum cymosum*, *Rubus multibracteatus*, *Solanum nigrum* , *Dendrocalamus hamiltonii*, *Arundo donax* , and *Dillenia indica* L (Table 1). The remaining candidate species were retained in Table 1 to document the initial screening process but were not included in the final elephant habitat model.

**TABLE 1:** Performance and screening status of MaxEnt models for 15 candidate food‐plant species of Asian elephants.

Number	Botanical name	Abbreviation	ROC (AUC)	Used in final model	Evidence basis
1	*Fagopyrum cymosum*	*F. cymosum*	0.891	Yes	Diet record + regional occurrence
2	*Rubus multibracteatus*	*R. multibracteatus*	0.868	Yes	Diet record + regional occurrence
3	*Solanum nigrum*	*S. nigrum*	0.861	Yes	Diet record + regional occurrence
4	*Dendrocalamus hamiltonii*	*D. hamiltonii*	0.853	Yes	Diet record + regional occurrence
5	*Arundo donax*	*A. donax*	0.842	Yes	Diet record + regional occurrence
6	*Dillenia indica* L.	*D. indica*	0.777	Yes	Diet record + regional occurrence
7	*Mallotus barbatus*	*M. barbatus*	0.768	No	Diet record + regional occurrence
8	*Aporosa yunnanensis*	*A. yunnanensis*	0.732	No	Diet record + regional occurrence
9	*Cratoxylum cochinchinense*	*C. cochinchinense*	0.722	No	Diet record + regional occurrence
10	*Thysanolaena latifolia*	*T. latifolia*	0.709	No	Diet record + regional occurrence
11	*Phyllanthus emblica*	*P. emblica*	0.703	No	Diet record + regional occurrence
12	*Psidium guajava*	*P. guajava*	0.701	No	Diet record + regional occurrence
13	*Macaranga denticulata*	*M. denticulata*	0.7	No	Diet record + regional occurrence
14	*Bombax ceiba*	*B. ceiba*	0.681	No	Diet record + regional occurrence
15	*Cinnamomum caudatum*	*C. caudatum*	0.5	No	Diet record + regional occurrence

#### 2.1.2 Environmental Variable Screening

##### 2.1.2.1 Screening of Climatic and Topographic Factors

To optimize model performance and avoid overfitting from multicollinearity, correlation analyses were performed on 22 climatic and topographic variables using ENMTools. Variable selection was further refined based on the ecological habits of Asian elephants, eliminating covariance interference to ensure the final predictors accurately represented the species' ecological requirements (Zhao, Wei, et al. 2025; Li, Wang, et al. 2025). We used |r| > 0.7 as a conservative threshold to identify highly collinear predictors, following common practice in SDM studies (e.g., Li, Luo, et al. 2024; Um et al. 2025; Figure S1). Retaining variables with lower pairwise correlations reduces multicollinearity and improves the interpretability and transferability of model coefficients, while still capturing the key climatic gradients that are ecologically relevant for Asian elephants. Because food‐plant distributions may partly reflect underlying climatic gradients, potential redundancy between the retained food‐plant resource layers and abiotic predictors was also considered before final model construction. Food‐plant variables were therefore interpreted as climate‐mediated proxies for potential food‐resource availability rather than as independent causal drivers of Asian elephant habitat suitability.

A combinatorial analysis was conducted on the six selected food‐plant variables. A total of 22 plant‐variable combinations were generated using the combinatorial formula $C\left(n,k\right)=\frac{k}{k\times \left(n-k\right)}$, where *n* = 6 and *k* was set to 4, 5, or 6. Model performance and ecological interpretability were highest in combinations that included *D. indica* suggesting that this species provided important resource‐related information for predicting elephant habitat suitability. We therefore compared two modeling scenarios: a baseline model excluding food‐plant variables and an extended model incorporating the six retained food‐plant variables. This comparison allowed us to quantify how food‐plant resource layers modified predicted habitat suitability, habitat area, and spatial configuration.

##### 2.1.2.2 Expert‐Informed Prior

**FIGURE 2:**
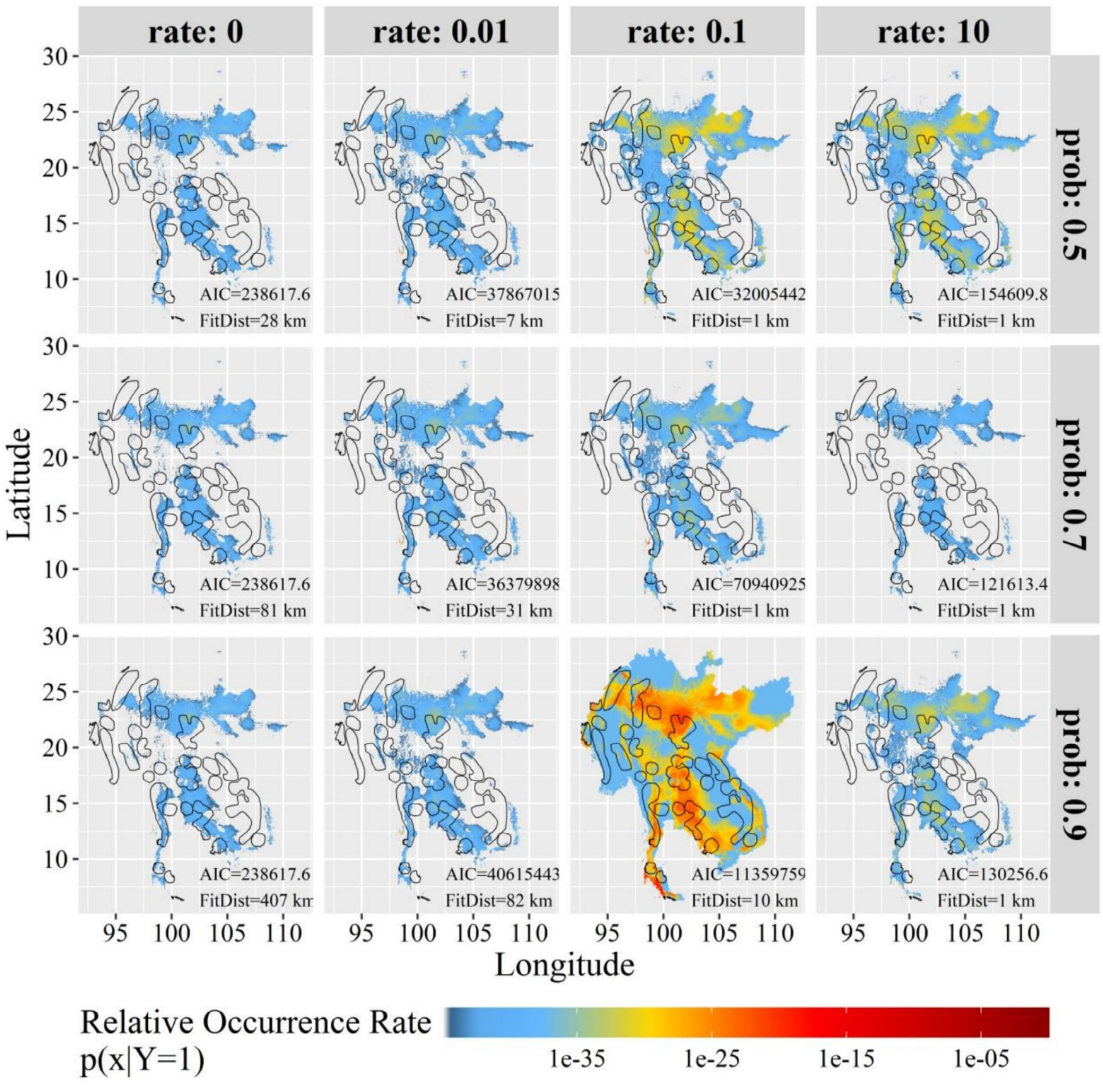
Integration of expert‐supported range information, historical range information, and recent occurrence records for Asian elephant species distribution modeling.

To reduce spatial sampling bias in occurrence records and improve the ecological plausibility of model predictions, we constructed an expert‐informed prior layer by integrating historical range information, recent monitoring records, and expert‐drawn distribution boundaries for Asian elephants (Merow et al. 2017; Sandel et al. 2025; Araujo et al. 2025; Figure 2). Following the expert‐map offset framework of Merow et al. (2017), this range information was converted into a continuous spatial prior using the bossMaps package in R. In this framework, expert maps provide broad‐scale information on the plausible distributional limits of a species, whereas occurrence records provide finer‐scale information for estimating environmental associations. Therefore, the expert‐informed prior was used to complement occurrence‐only data and to reduce unrealistic extrapolation into areas outside the known and expert‐supported Asian elephant range (Merow et al. 2017; Figure 2). The expert‐informed prior was used only as a spatial correction layer to reduce sampling bias and unrealistic extrapolation outside the expert‐supported range. It was not interpreted as an ecological predictor of Asian elephant habitat suitability.

Candidate prior parameter combinations were compared using AIC, and the combination with the lowest AIC was selected for the final bias‐corrected MaxEnt model. The optimal combination was Pin=0.70 and r=10. Here, Pin=0.70 indicates that 70% of the cumulative prior probability was assigned to the expert‐supported Asian elephant range, reflecting moderate confidence in the expert range information. The decay parameter *r* = 10 indicates a steep spatial decline in prior probability outside the expert range boundary, meaning that areas farther from the known range were assigned lower prior expectation unless supported by the occurrence–environment relationship. The resulting expert‐informed prior was incorporated into MaxEnt as a spatial offset. Thus, this layer was interpreted as prior distributional knowledge for bias correction and range constraint, rather than as an independent causal driver of Asian elephant habitat suitability.

### 2.2 MaxEnt Model

#### 2.2.1 MaxEnt Model

The Maximum Entropy (MaxEnt) model estimates species habitat suitability by relating known occurrence records to environmental constraints under the principle of maximum entropy. Owing to its robustness and predictive performance, including cases with limited occurrence records, it is extensively applied in species distribution modeling (Zhang et al. 2024; Li et al. 2026). The MaxEnt model requires careful parameterization, particularly for the feature combination (FC) and regularization multiplier (RM). Fine‐tuning these parameters is a critical step to reduce overfitting and improve model performance and transferability (Zhang et al. 2024; Li, Wang, et al. 2025). Parameter optimization for the MaxEnt model was carried out using the ENMeval package in R (Li et al. 2026). This process involved testing five distinct feature classes: linear (L), quadratic (Q), hinge (H), product (P), and threshold (T). Six feature combinations (H, L, LQ, LQH, LQHP, and LQHPT) were tested. The regularization multiplier was varied across 40 values from 0.1 to 4.0 in increments of 0.1. Among all candidate models, the one with the minimum corrected Akaike Information Criterion (AICc) was selected as the optimal model (de Moll et al. 2026). The predictive performance of the selected model was then evaluated using the receiver operating characteristic (ROC) curve, the area under the curve (AUC), and the True Skill Statistic (TSS) (Zhang et al. 2024; Li, Wang, et al. 2025). Model discrimination was assessed using AUC, which ranges from 0.5 (random prediction) to 1.0 (perfect discrimination). Following the classification by Li, Wang, et al. (2025), predictive performance was rated as poor (0.5–0.6), fair (0.6–0.7), good (0.7–0.8), very good (0.8–0.9), or excellent (> 0.9) (Li, Li, et al. 2025). The True Skill Statistic (TSS) was derived from the confusion matrix to quantify the agreement between observed and predicted distributions (de Moll et al. 2026). It was calculated using the maximum training sensitivity plus specificity threshold and ranges from −1 to 1, where values closer to 1 indicate better model performance (Merow et al. 2017; Li, Li, et al. 2025).

#### 2.2.2 Optimization of the MaxEnt Model

**FIGURE 3:**
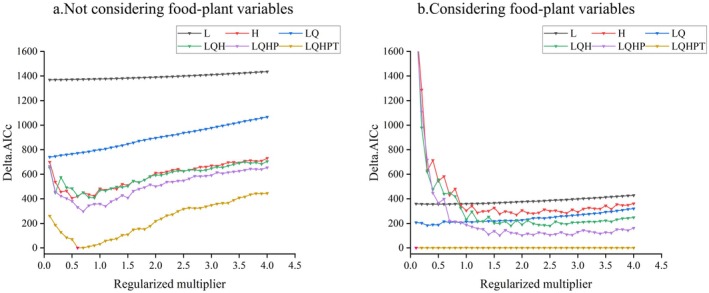
Evaluation and selection of MaxEnt model parameters using the ENMeval framework. (a) Not considering food‐plant variables. (b) Considering food‐plant variables.

The ENMeval analysis generated 480 candidate models for the two scenarios with and without food‐plant variables. Following standard model selection criteria, the model with a delta AICc value of 0 was identified as optimal. Notably, in both scenarios, the top‐performing model shared the same configuration: an LQHPT feature combination and a regularization multiplier of 0.6. This configuration showed high predictive performance while reducing model overfitting (Figure 3).

### 2.3 Current and Future Distribution Patterns of Suitable Asian Elephant Habitats

The optimized species distribution model was constructed using Asian elephant occurrence records and the selected environmental predictors in the MaxEnt algorithm, with the optimal feature combination and regularization multiplier as determined by the ENMeval analysis (Li, Li, et al. 2025). Model runs were configured with ten replicates and 780 randomly selected background points, corresponding to the number of occurrence records. A random subset of 75% of the records was used for model training, and the remaining 25% was retained for validation. The relative importance of environmental variables in the model was assessed using a Jackknife test, which evaluates the change in model gain when each variable is used alone or omitted. The relationship between predictor variables and predicted habitat suitability for Asian elephants was examined using response curves. All model predictions were output in logistic format, as recommended for species distribution modeling (Song et al. 2021; Li, Luo, et al. 2024). The model's logistic output was converted into raster format (Lai et al. 2022). Habitat suitability was then classified using the “Reclassify” function, with threshold values defined by combining IPCC reporting criteria (Um et al. 2025) and current Asian elephant distribution patterns. The reclassification scheme was defined as follows: Unsuitable (0–0.06), Low suitability (0.06–0.192), Medium suitability (0.192–0.459), and High suitability (0.459–1.0).

### 2.4 Changes in the Ecological Niche of Asian Elephants

We evaluated differences in the predicted environmental niche space of Asian elephants using a comparative framework with and without food‐plant resource variables, following prior niche studies (Um et al. 2025). To characterize the environmental niche space, we performed an environmental principal component analysis (PCAenv) on the environmental predictors associated with predicted Asian elephant habitat suitability. The similarity of niches under the two scenarios was evaluated using the Ecospat package. Specifically, a null distribution of niche overlap was constructed from 1000 randomization simulations. Statistical significance was assessed by comparing the observed niche overlap with this null distribution; rejection of the null hypothesis (*p* < 0.05) was interpreted as statistical support for niche differentiation (Li, Mo, et al. 2024; Dong et al. 2026).

### 2.5 Species Interactions

Piecewise structural equation modeling (SEM) was used to examine hypothesized statistical associations among climatic variables, topographic variables, food‐plant variables, observed Asian elephant activity records, and predicted *E. maximus* habitat suitability along latitudinal gradients (Hu et al. 2024; Paterno et al. 2024; Xu et al. 2025). Following an a priori conceptual framework, we specified hypothesized pathways from climatic and topographic variables to food‐plant variables, and from climatic, topographic, food‐plant variables, and observed Asian elephant activity records to predicted *E. maximus* habitat suitability (Song and Tang 2025). Observed Asian elephant activity records were used as occurrence information and were not interpreted as independent environmental predictors. Separate SEMs were fitted for low‐ and high‐latitude regions and for different time periods to evaluate whether these associations varied across regional climate and vegetation gradients. Although the final retained pathways differed among panels, all SEMs were developed under the same conceptual framework. Before analysis, all continuous variables were standardized to allow comparison of standardized path coefficients. Following previous piecewise SEM studies, initial models were simplified by removing unsupported non‐significant pathways while retaining ecologically meaningful and statistically supported paths (Hu et al. 2024). Model fit was evaluated using Fisher's C statistic, degrees of freedom, associated *p*‐values, and marginal *R* ^2^ values for component models. A non‐significant Fisher's C test was interpreted as indicating acceptable model fit and no major missing pathways. Because both MaxEnt and SEM are correlative approaches, SEM pathways were interpreted as hypothesized statistical associations rather than direct causal effects.

## 3 Results

### 3.1 Model Prediction Performance

**FIGURE 4:**
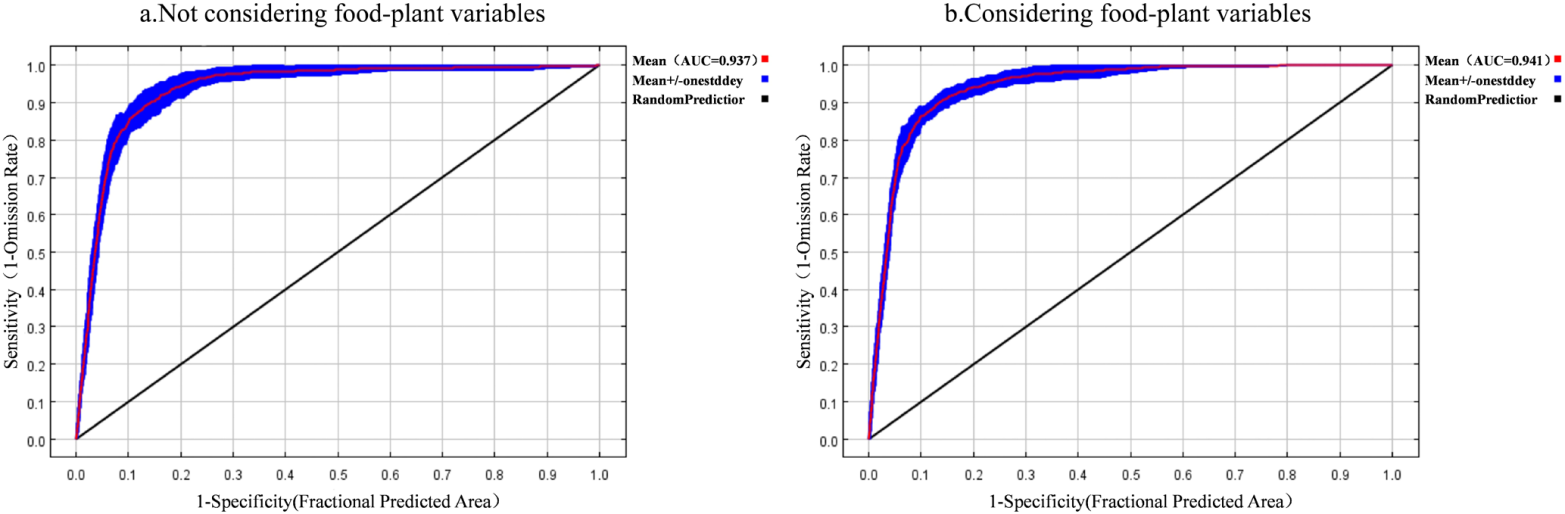
Evaluation of model prediction performance using receiver operating characteristic (ROC) curves. (a) Not considering food‐plant variables. (b) Considering food‐plant variables.

Based on ROC curve evaluation, the AUC values reached 0.937 and 0.941 for the baseline and food‐plant‐resource models, respectively, indicating excellent predictive performance and strong model discrimination ability. Comparative analysis further showed that the model incorporating food‐plant variables had a slightly higher AUC value, suggesting a modest improvement in predictive performance for the spatial assessment of Asian elephant habitat suitability (Figure 4).

### 3.2 Analysis of Environmental Variables

The results showed that when food‐plant resource variables were excluded, isothermality had the highest percent contribution (35.7%), followed by precipitation of the driest quarter (18.5%). In contrast, other predictors, including aspect and precipitation of the coldest quarter, had relatively low contributions to the model. When food‐plant variables were incorporated, the model results changed markedly. In the model incorporating food‐plant resource variables, the expert‐informed prior showed the highest contribution value (30.4%), reflecting the influence of spatial correction and range constraint. Among ecological predictors, *D. indica* had the highest percent contribution (17.3%). Our results suggest that food‐plant resource layers provided additional predictive information for habitat suitability. When food‐plant resource variables were incorporated, the relative contribution of the key climatic variable, isothermality, decreased markedly from 50.3% to 20.5%. This suggests a lower relative dominance of climatic predictors and a more multi‐factor predictor structure, in which climate remained an important, but not exclusive, predictor (Table 2).

**TABLE 2:** Contribution rates of environmental predictors in model simulations under two scenarios: With and without food‐plant variables.

a. Not considering food‐plant variables			b. Considering food‐plant variables		
Variable	Percent contribution/%	Permutation importance/%	Variable	Percent contribution/%	Permutation importance/%
Isothermality	35.7	50.3	Expert	30.4	8.8
Precipitation of Driest Quarter	18.5	16.2	*D. indica*	17.3	13
Precipitation of Wettest Quarter	17.8	18.1	Isothermality	15.3	20.5
Aspect	12	4.1	Slope	10.2	7
Precipitation of Coldest Quarter	8.4	4.9	Precipitation of Wettest Quarter	8.4	14.7
			Precipitation of Coldest Quarter	7.2	17.5
			*S. nigrum*	2.2	4.9
			Aspect	2.2	1.9
			*F. cymosum*	1.6	3.8
			*D. hamiltonii*	1.4	0.9
			Precipitation of Driest Quarter	1.4	2.1
			*Arundo donax*	1.3	1.8
			*R. multibracteatus*	0.9	1.9
			Elevation	0.3	1.1

**FIGURE 5:**
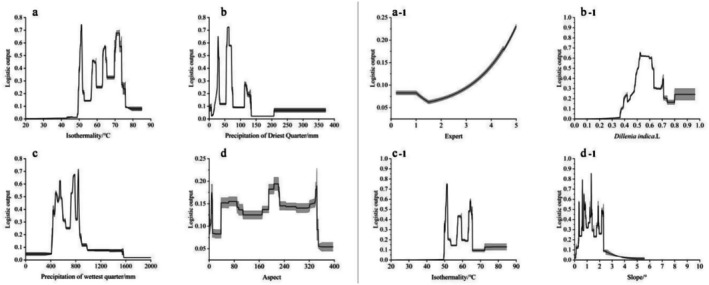
Comparison of response curves between baseline and food‐plant‐resource models for Asian elephant habitat suitability. (a–d) Represent the model excluding food‐plant resource variables, whereas (a‐1–d‐1) represent the model incorporating food‐plant resource variables. The selected ecological predictors include climatic, topographic, and food‐plant resource layers, whereas the expert‐informed prior is shown as a spatial offset rather than an ecological predictor. The black lines indicate mean logistic output, and the gray bands represent variation among model replicates.

Although the expert‐informed prior appeared in the MaxEnt output, it was included as a correction layer rather than as an ecological predictor. Therefore, its contribution value was used only to indicate the strength of spatial correction and was not compared ecologically with climatic, topographic, or food‐plant variables. When food‐plant resource variables were excluded, isothermality was associated with the highest predicted habitat suitability at intermediate isothermality values (40–60). Precipitation during the driest quarter (~150 mm) and the wettest quarter (1000–1500 mm) were also associated with higher predicted habitat suitability. Our analysis indicated higher predicted habitat suitability within these identified precipitation intervals, suggesting the importance of the precipitation regime. Meanwhile, the influence of slope was negligible in comparison, manifesting only as a slight increase in suitability on very gentle slopes of 0°–5° (Figure 5a–d).

After integrating food‐plant resource variables, the response curve for the expert‐informed prior indicated that areas with higher prior support were assigned higher predicted suitability, reflecting its role as a spatial offset rather than an ecological response variable. The suitability response associated with the *D. indica* layer was highest within a predicted distribution probability range of 0.2–0.6, suggesting the relevance of specific food resources. Concurrently, the suitability response to isothermality shifted slightly toward lower variability, suggesting possible covariation between food‐plant resource layers and climatic gradients. While the overall influence of slope diminished, it maintained a notable effect in areas of low inclination. In addition, the contribution from precipitation during the wettest quarter remained relatively important, further suggesting the continued relevance of precipitation patterns in predicting habitat suitability. In summary, the inclusion of food‐plant resource variables was associated with a more balanced predictor structure: it reduced the relative dominance of climatic predictors, highlighted potential associations between food resources and environmental gradients, and modestly improved predictive performance for Asian elephant habitat suitability (Figure 5a‐1–d‐1).

### 3.3 Current and Future Patterns of the Distribution of Suitable Areas

**FIGURE 6:**
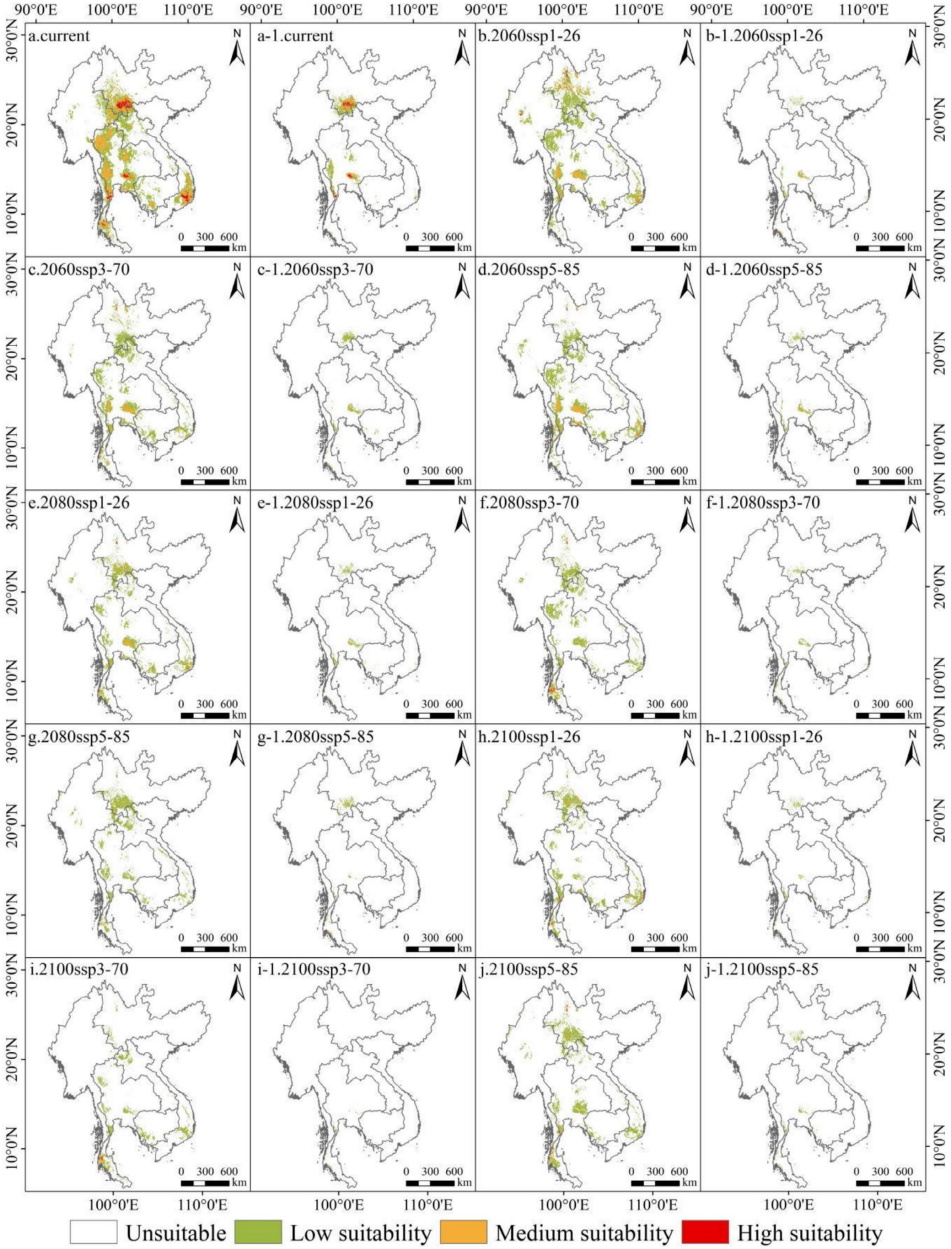
Current and projected future distributions of suitable habitat for *E. maximus* under models excluding and incorporating food‐plant variables. (a–j) show predictions from the model excluding food‐plant variables, whereas (a‐1–j‐1) show predictions from the model incorporating food‐plant variables.

**FIGURE 7:**
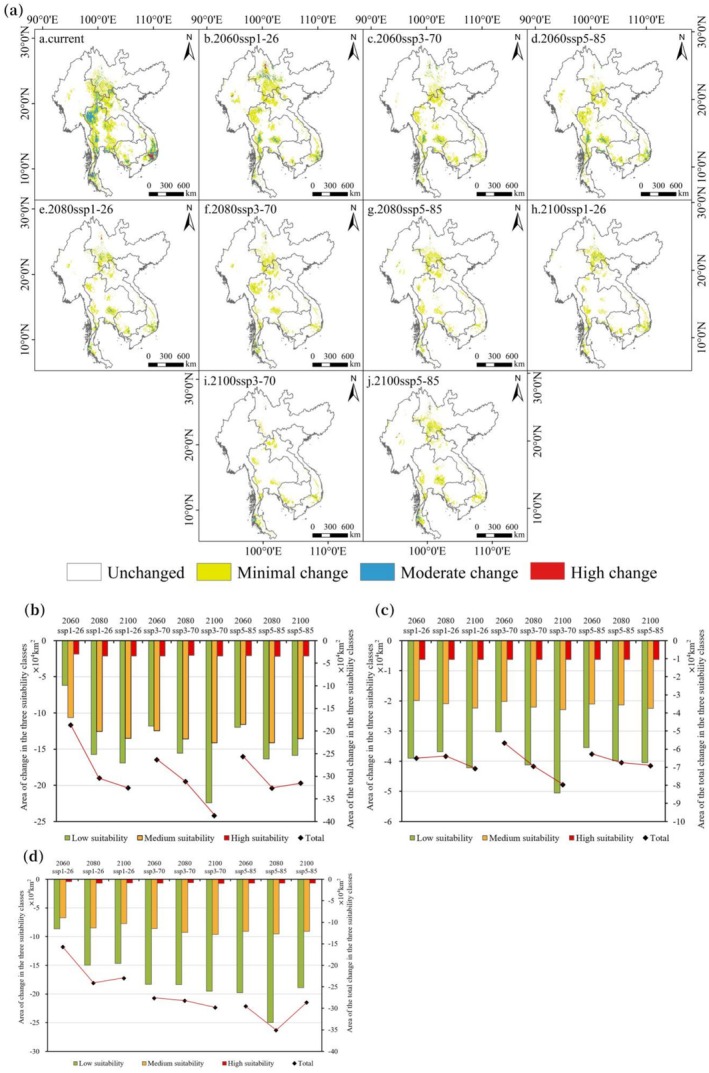
(a) Spatial changes in predicted habitat suitability after incorporating food‐plant variables. (b–d) Changes in predicted suitable habitat area for *E. maximus* under future climate scenarios: (b) without food‐plant variables, (c) with food‐plant variables, (d) differences between frameworks.

The predicted suitable habitat for *E. maximus* differed markedly between the models excluding and incorporating food‐plant variables (Figure 6 and Figure 7b,d). In the model excluding food‐plant variables, suitable habitats were broadly distributed across mainland Southeast Asia and southern Yunnan, China, with low‐ and medium‐suitability patches extending over a relatively wide geographic range. Under current conditions, the total suitable area reached 465,862.93 km^2^. After food‐plant variables were incorporated, suitable habitats became substantially more restricted and spatially concentrated, mainly occurring in southern Yunnan and parts of mainland Southeast Asia. The current total suitable area decreased to 82,903.22 km^2^, representing an 82.2% reduction. High‐suitability habitat also decreased from 21,836.10 km^2^ to 6197.79 km^2^.

Future projections showed a general contraction of suitable habitat under both modeling frameworks, but the contraction was more pronounced after food‐plant variables were incorporated (Figure 6). In the model excluding food‐plant variables, future suitable habitats remained relatively extensive under several scenarios, although low‐, medium‐, and high‐suitability areas generally declined relative to current conditions. By contrast, in the model incorporating food‐plant variables, future suitable habitats were consistently smaller and more fragmented. After food‐plant variables were incorporated, total suitable habitat declined from the 2060s to the 2100 s under all SSP scenarios. Under SSP1‐2.6, total suitable area decreased from 17,937.16 km^2^ in the 2060s to 12,291.87 km^2^ in the 2100 s. Under SSP3‐7.0, it declined more sharply from 26,452.22 km^2^ to 3635.11 km^2^. Under SSP5‐8.5, it decreased from 20,221.06 km^2^ to 13,907.94 km^2^. High‐suitability habitat was greatly reduced or nearly absent under most future scenarios after food‐plant variables were included.

The area‐change statistics supported this spatial pattern (Figure 7a,d). Relative to current conditions, the reduction in suitable habitat was mainly associated with decreases in low‐ and medium‐suitability areas, whereas high‐suitability areas remained limited under future scenarios. The contrast between the two modeling frameworks showed that the model excluding food‐plant variables consistently predicted larger suitable areas than the model incorporating food‐plant variables. This indicates that broad climatic suitability does not necessarily represent potentially usable habitat when food‐resource availability is considered.

Overall, incorporating food‐plant variables refined the habitat suitability predictions by distinguishing broad climate‐based suitability from more restricted resource‐based suitability. These results suggest that models excluding food‐plant variables may overestimate potentially usable habitat for *E. maximus* . Areas showing moderate or high changes should be considered priority zones for field verification, food‐plant restoration, and habitat management because they may represent mismatches between climatic suitability and resource‐supported habitat suitability.

### 3.4 Ecological Niche

**FIGURE 8:**
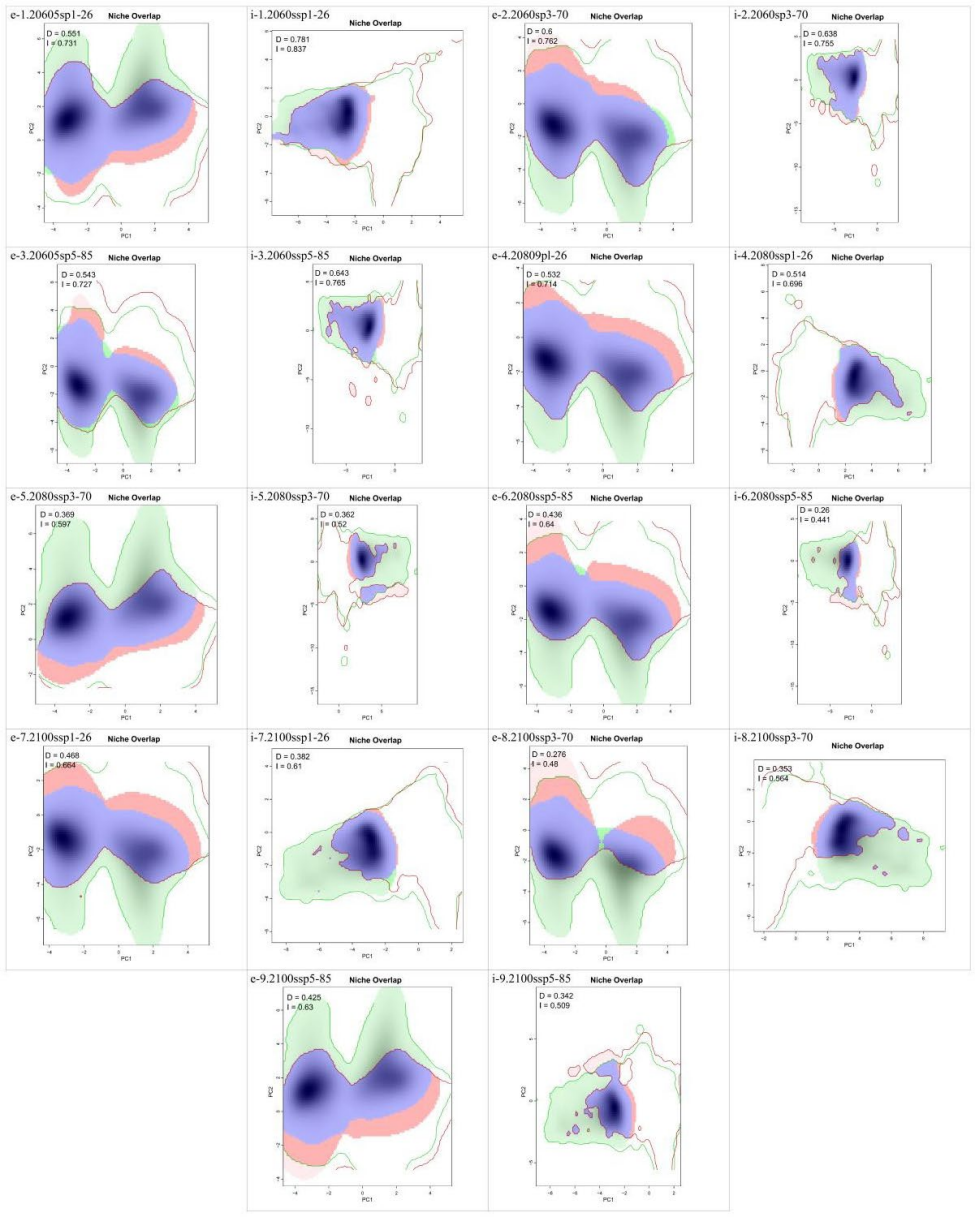
Ecological niche dynamics of Asian elephants under scenarios excluding and incorporating food‐plant resource variables. (e‐1–e‐10) show predictions from the model excluding food‐plant resource variables, whereas (i‐1–i‐10) show predictions from the model incorporating food‐plant resource variables.

Ecological niche overlap varied across future climate scenarios and between the two modeling frameworks (Figure 8). In the model excluding food‐plant variables, niche overlap was generally moderate, with Schoener's D ranging from 0.270 to 0.600 and the I index ranging from 0.480 to 0.762. Relatively higher overlap values were observed in the 2060s under SSP1‐2.6 and SSP3‐7.0, whereas lower overlap values occurred in the 2100 s, especially under SSP3‐7.0. These results suggest that, when food‐plant variables were excluded, the predicted ecological niche of Asian elephants remained relatively broad, although overlap decreased under some future climate scenarios.

After incorporating food‐plant variables, the pattern of niche overlap changed noticeably. Schoener's *D* ranged from 0.260 to 0.810, and the I index ranged from 0.441 to 0.837. In several scenarios, niche overlap increased after food‐plant variables were included, indicating a stronger concentration of predicted suitable conditions in environmental space. However, this pattern was not consistent across all scenarios. For example, relatively high overlap was observed in the 2060s under SSP1‐2.6, whereas lower overlap values occurred in the 2080s and 2100 s under higher‐emission scenarios. This indicates that the inclusion of food‐plant variables made the predicted niche pattern more sensitive to differences among future climate scenarios.

Overall, the ecological niche analysis showed that food‐plant variables altered the distribution of predicted suitable conditions in environmental space. Rather than simply increasing niche overlap, the inclusion of food‐plant variables refined the predicted niche by concentrating suitability in areas where climatic conditions and potential food resources overlapped. These results suggest that food‐plant availability can help distinguish broad climatic suitability from more restricted resource‐based suitability for Asian elephants under future climate change.

### 3.5 Latitudinal SEM Analysis

Model fit statistics for the eight piecewise SEMs are summarized in Table 3. All SEMs showed acceptable model fit, with Fisher's C values ranging from 1.16 to 7.58 and *p* values ranging from 0.108 to 0.885. Because all Fisher's C tests were non‐significant, the fitted SEMs did not indicate major missing pathways. Although the retained pathways differed among panels, all models were developed under the same conceptual framework and evaluated using the same model‐fit criteria (Table 3).

**TABLE 3:** Model fit statistics for piecewise SEMs across latitudinal zones and future periods.

Panel	Period	Latitude zone	Fisher's C	df	*p*	Model fit
1	Current	Low latitude	2.03	4	0.73	Acceptable
2	Current	High latitude	4.2	2	0.123	Acceptable
3	2060s	Low latitude	1.16	4	0.885	Acceptable
4	2060s	High latitude	7.58	4	0.108	Acceptable
5	2080s	Low latitude	1.88	4	0.758	Acceptable
6	2080s	High latitude	7.58	4	0.108	Acceptable
7	2100 s	Low latitude	1.45	4	0.835	Acceptable
8	2100 s	High latitude	2.13	2	0.34	Acceptable

**FIGURE 9:**
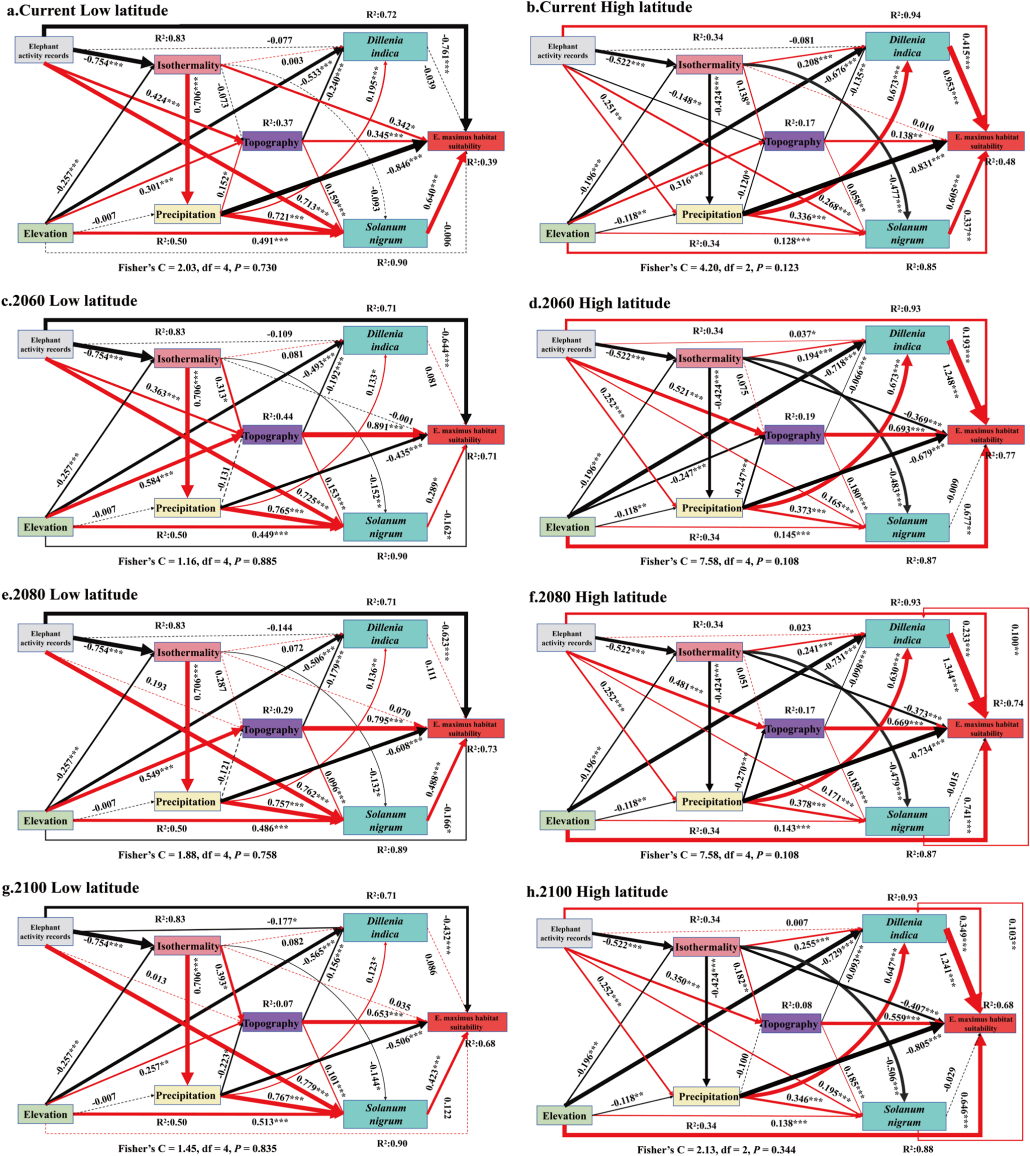
Interaction pathways between Asian elephants and plant factors derived from structural equation modeling.

We used structural equation modeling to examine statistical associations among climatic, topographic, food‐plant variables, and elephant habitat suitability. A significant negative association was found between elephant habitat suitability and both precipitation (Std. Estimate = −0.846, *p* < 0.001) and latitude (Std. Estimate = −0.761, *p* < 0.001) at low latitudes. Conversely, significant positive associations were identified for isothermality (Std. Estimate = 0.342, *p* < 0.05), topographic factors (Std. Estimate = 0.345, *p* < 0.05), and the distribution of *S. nigrum* (Std. Estimate = 0.640, *p* < 0.05). Furthermore, latitude and elevation were associated with the pathways linking isothermality, topography, and *S. nigrum* to predicted elephant habitat suitability. While the key food plant *D. indica* was strongly associated with elevation (Std. Est. = −0.533), precipitation (Std. Est. = 0.195), and topography (Std. Est. = −0.240) (all *p* < 0.001), it was not directly associated with predicted Asian elephant habitat suitability (Figure 9).

The variance explained (*R* ^2^) in the low‐latitude model was 0.83 for isothermality, 0.50 for precipitation, 0.37 for slope_aspect, 0.72 for *D. indica* , and 0.90 for *S. nigrum* . Collectively, these factors explained 39% (*R* ^2^ = 0.39) of the variance in Asian elephant distribution, indicating that climatic and food‐plant variables jointly contributed to the statistical explanation of elephant habitat suitability in low‐latitude regions (Figure 9).

In contrast to low latitudes, the high‐latitude model revealed a distinct pattern of associations, with *D. indica* showing the strongest positive association with predicted Asian elephant habitat suitability (Std. Estimate = 0.953, *p* < 0.01). This was followed by significant associations with precipitation (Std. Estimate = −0.831, *p* < 0.001) and *S. nigrum* (Std. Estimate = 0.605, *p* < 0.001). The high explanatory power of the food‐plant resource variables (*R* ^2^ = 0.94 for *D. indica* , *R* ^2^ = 0.85 for *S. nigrum* ) collectively contributed to the model's ability to explain 48% of the distribution variance (*R* ^2^ = 0.48), suggesting that food‐plant resource variables were closely associated with elephant habitat suitability in high‐latitude regions (Figure 9).

The structural equation models for current and future periods showed acceptable fit at both low and high latitudes. Across the eight SEMs, Fisher's C ranged from 1.16 to 7.58, df ranged from 2 to 4, and *p* values ranged from 0.108 to 0.885. Because all Fisher's C tests were non‐significant, no major missing pathways were detected. Although the retained pathways differed among panels, all models were developed under the same conceptual framework and evaluated using the same model‐fit criteria. The models consistently explained a high proportion of variance in future elephant distribution (*R* ^2^ = 0.68–0.73 at low latitudes; *R* ^2^ = 0.68–0.77 at high latitudes). Crucially, the explanatory power for the key vegetation resources ( *S. nigrum* and *D. indica* .) was even higher (*R* ^2^ = 0.89–0.90 at low latitudes; *R* ^2^ = 0.93 at high latitudes), indicating their strong explanatory relevance in the fitted SEMs across future scenarios (Figure 9).

At low latitudes, projections from the 2060s to the 2100 s consistently showed that precipitation had a significant and persistent negative association with elephant habitat suitability (Std. Estimate: −0.435 to −0.644, *p* < 0.001). In contrast, the positive association of *S. nigrum* remained stable across all periods. However, the association of topographic factors with habitat suitability exhibited a declining trend over time (Std. Estimate decreasing from 0.891 to 0.653). This temporal pattern suggests that food‐plant variables retained explanatory value as the association between terrain and habitat suitability weakened over time (Figure 9).

At high latitudes, the association of isothermality differed markedly from low‐latitude patterns, showing an increasingly negative association over time (Std. Estimate = −0.369 to −0.407), alongside a similarly strengthening negative association with precipitation (Std. Estimate = −0.679 to −0.805). In contrast, the association between observed Asian elephant activity records and elephant habitat suitability increased over time, while the association with terrain decreased (Std. Estimate = 0.193–0.349). Throughout all future periods, *D. indica* remained strongly and positively associated with elephant habitat suitability, suggesting that this food‐plant variable provided important resource‐related information in the habitat suitability model (Std. Estimate≈1.3, *p* < 0.001). Furthermore, *S. nigrum* did not show a direct association with elephant habitat suitability, but it was associated with *D. indica*, suggesting that these food‐plant variables may represent related resource conditions (Figure 9). These results provide spatial information for developing latitude‐specific conservation actions. At low latitudes, the focus should be on managing hydrological regimes and topographic‐soil conditions to support grass‐dominated forage. At high latitudes, conservation planning should prioritize the protection and restoration of key food resources, especially *D. indica* , which was strongly associated with habitat suitability in the SEM (Figure 9). This latitudinal framework enables more precise and effective habitat management.

## 4 Discussion

### 4.1 Analysis of Changes in Suitable Areas

While the predicted distribution of Asian elephant habitat is associated with complex climate–vegetation relationships, conventional models have predominantly emphasized hydrothermal conditions, consistent with the species' generalist ecological strategy (Baskaran et al. 2011). Our MaxEnt results supported the importance of climatic predictors when food‐plant resource variables were excluded; however, the inclusion of key food‐plant layers changed the relative contribution pattern. The inclusion of species such as *D. indica* suggested that food‐plant resource layers provided additional predictive information, although this information may partly reflect climate‐mediated vegetation patterns rather than independent plant effects. This pattern is consistent with observations that spatially concentrated food resources, including species such as *S. nigrum* , may be associated with elephant habitat use and local “food hotspots” (Abir et al. 2025).

As generalist megaherbivores, Asian elephants consume grasses, palms, bamboo shoots, wild plantain, and woody plant leaves and fruits (Jiang et al. 2019; Abir et al. 2025). In the Nangunhe National Nature Reserve, Yunnan, grasses (Poaceae) accounted for 47.69% of the recorded diet, followed by Moraceae (21.25%) and Plantaginaceae (11.24%) (Liu et al. 2016; Wu et al. 2021; Abir et al. 2025). In southwestern Yunnan, expansion of rubber and tea plantations has reduced the availability of wild plants important for elephant nutrition (Liu et al. 2025). Reduced natural forage may be associated with increased elephant use of agro‐ecosystems, where crops such as rice, maize, and sugarcane provide concentrated, energy‐rich food resources (Wang 2022; Xu 2024). This behavioral transition highlights their adaptability to anthropogenic landscapes, but it is also associated with increased human–elephant conflict and potential ecological traps (Xu 2024; Figure S2).

Incorporating food‐plant resource variables provided a more resource‐informed modeling perspective than models relying only on abiotic predictors. Our results suggest that models excluding food‐plant resource variables may overestimate predicted suitable habitat, whereas integrating food‐plant resource layers produced a more constrained prediction. This indicates that food‐resource availability is an important predictor often overlooked in conventional distribution models. Because food‐plant distributions are also shaped by climatic and topographic conditions, food‐plant resource variables should be interpreted as climate‐mediated resource proxies rather than independent causal drivers of Asian elephant habitat suitability. Given that climate change is projected to alter vegetation distributions, such models may support more resource‐informed assessments of future habitat change and conservation planning.

### 4.2 Climate–Resource Associations and Species Interactions

Our piecewise structural equation modeling (SEM) across latitudinal gradients suggested spatiotemporal differences in the associations among climatic, topographic, and food‐resource predictors of Asian elephant habitat suitability. A key finding was the negative association between precipitation‐related predictors and predicted habitat suitability, consistent with studies linking precipitation to vegetation dynamics and habitat use (Bohrer et al. 2014; Budhathoki et al. 2023). This pattern may reflect changes in vegetation productivity and wetland hydrology under altered precipitation regimes. Isothermality was an important predictor, especially at higher latitudes, consistent with its role as a broad‐scale distribution predictor (María and Carlos 2012). Temperature variability may alter plant phenology and food‐resource availability, which is relevant to megafauna responses to climate extremes (Sunday et al. 2014). These associations are also consistent with the species' use of resource‐rich, topographically gentle river valleys (He et al. 2023). The relative importance of key food plants varied along latitudinal gradients. *D. indica* was important at high latitudes, whereas *S. nigrum* and bamboo resources contributed more strongly in low‐latitude regions (Wang et al. 2021). Nutritional analyses indicate that *D. indica* fruits provide high energy and may support seed dispersal and mutualistic plant–elephant interactions (Saikia et al. 2023). Regional monitoring also reports that climate warming and forest degradation are associated with habitat fragmentation and food‐resource depletion (Kumar et al. 2023). These results suggest different conservation priorities across latitudes. Low‐latitude regions should consider managing precipitation‐related habitat stress and conserving food resources such as *S. nigrum* , whereas high‐latitude regions should consider safeguarding fruit‐bearing trees such as *Dillenia indica* L. and enhancing habitat connectivity. Predictive dispersal modeling may help delineate ecological corridors and buffer zones under scenarios such as SSP1‐2.6 and SSP3‐7.0, supporting long‐term connectivity and resilience (Guarnieri et al. 2024; Bai et al. 2022).

### 4.3 Ecological Niche Dynamics and Insights Into Conservation

The niche analysis suggests that food‐plant resource variables are relevant to the predicted environmental niche space of Asian elephants. Models incorporating food‐plant resource variables showed more concentrated suitability patterns in areas with higher predicted food‐resource availability. This pattern is consistent with the species' generalist foraging strategy and behavioral plasticity across heterogeneous landscapes (Fernando and Pastorini 2011; Zhang et al. 2024). However, these results should not be interpreted as evidence that food plants alone define the ecological niche of Asian elephants. Instead, food‐resource layers provide additional spatial information that complements climatic and topographic predictors.

Predicted niche overlap varied under future climate scenarios, increasing where climatic suitability and predicted food‐resource availability were more closely aligned, and decreasing where resource‐supported suitability became more restricted. These dynamics may be linked to climate‐related changes in vegetation distribution, through which regional differences in precipitation and temperature affect the spatial availability of key food plants.

Overall, incorporating food‐plant resource variables modestly improved habitat suitability predictions and highlighted the relevance of food resources to the predicted environmental niche of Asian elephants (Pringle et al. 2023; Li, Mo, et al. 2024). Future conservation strategies should therefore integrate projected food‐resource availability into species management frameworks. We propose three priorities: protecting and restoring key food plants, enhancing habitat connectivity, and implementing adaptive management informed by projected climate scenarios. This integrated strategy may support the long‐term persistence of Asian elephant populations under climate and land‐use change (Abeysinghe 2016).

## 5 Conclusion

This study used a comparative habitat suitability modeling approach to evaluate how explicitly incorporating food‐plant resource layers modifies predictions of Asian elephant ( *E. maximus* ) habitat suitability under current and future climate scenarios. Compared with the model excluding food‐plant variables, the model incorporating food‐plant variables predicted a more restricted and spatially concentrated pattern of suitable habitat. Under current conditions, the predicted total suitable area decreased from 465,862.93 km^2^ to 82,903.22 km^2^ after food‐plant variables were included, indicating that climate‐dominated models may overestimate potentially usable habitat where key forage resources are limited or absent.

The expert‐informed prior was used as a correction layer rather than as an ecological predictor; therefore, the ecological interpretation of the model focused on climatic, topographic, and food‐plant variables. Food‐plant variables should not be interpreted as independent causal drivers that replace climatic or topographic predictors. Instead, they provide resource‐related information that complements abiotic predictors and helps distinguish broad climatic suitability from more restricted resource‐supported habitat suitability. The ecological niche analysis further showed that food‐plant variables altered the predicted environmental niche by concentrating suitability in areas where climatic conditions and potential forage resources overlapped.

Latitudinal SEM analysis suggested that the associations among climate, topography, food‐plant variables, observed elephant activity records, and predicted habitat suitability varied across regions and future periods. In low‐latitude regions, *S. nigrum* and terrain‐related variables showed stronger associations with predicted suitability, whereas *D. indica* provided important resource‐related information in high‐latitude regions. Because MaxEnt and SEM are correlative approaches, these patterns should be interpreted as statistical associations rather than direct causal effects.

Overall, these findings suggest that conservation planning for Asian elephants should move beyond climate‐based suitability alone. Priority should be given to areas where climatic suitability overlaps with potential food‐resource availability. Areas showing a mismatch between climatic suitability and food‐plant availability should be targeted for field verification, food‐plant restoration, and habitat connectivity planning under future climate change.

## Author Contributions

**Churui Li:** formal analysis (equal), resources (equal), writing – original draft (equal), writing – review and editing (equal). **Xiaorui Wang:** funding acquisition (equal), writing – review and editing (equal). **ZhenPing Qiang:** conceptualization (lead), funding acquisition (lead), methodology (lead), project administration (equal), supervision (lead). **Yongjing Tang:** methodology (equal), supervision (equal). **Song Yang:** investigation (equal), supervision (equal). **Chenglong Luo:** investigation (equal), resources (equal). **Xiaoyun Tu:** formal analysis (equal), supervision (equal). **Yuting Xia:** formal analysis (supporting), software (supporting), visualization (supporting). **Shuang Zhang:** formal analysis (supporting), supervision (supporting), visualization (supporting). **Lanzhong Zhang:** formal analysis (supporting), software (supporting), visualization (equal).

## Funding

The projects of the Natural Science Foundation of China (Grant No. 12163004), the Yunnan Fundamental Research Project (Grant No. 202301BD070001‐008, 202401AS070009), the Key Research and Development Project of Yunnan Province (Grant No. 202402AD080002‐5), and the Key Research and Development Program of Yunnan Province (Grant No.: 202503AP140040).

## Conflicts of Interest

The authors declare no conflicts of interest.

## Supporting information


**Data S1:** ece373793‐sup‐0001‐Supinfo01.docx.

**Data S2:** ece373793‐sup‐0002‐Supinfo02.docx.

**Data S3:** ece373793‐sup‐0003‐Supinfo03.docx.

## Data Availability

The data used in this paper, including environmental variables and species occurrence records, is available on the Open Science Framework. DOI: https://osf.io/8pehw/overview?view_only=254e86dee1144bbe9004b658a9630119.
