# Supplementary figures and images for: A Multi‐Factor Habitat Suitability Model for Asian Elephants in the Greater Mekong Subregion: Effects of Vegetation and Climate
Source: Ecol Evol. 2026 Jun 16;16(6):e73793. doi: 10.1002/ece3.73793 (PMC13270213; doi:10.1002/ece3.73793)

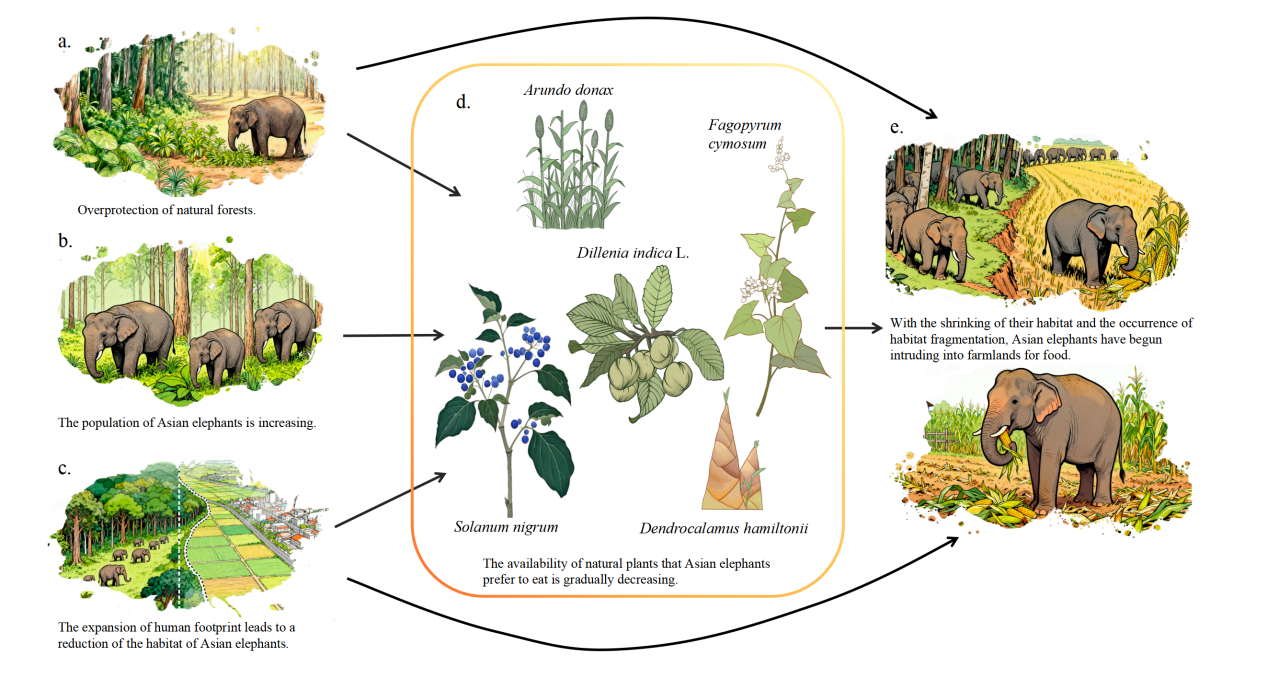

Supplement: Supplementary file 1 — Data S1: ece373793‐sup‐0001‐Supinfo01.docx. [file ECE3-16-e73793-s003.docx]

**
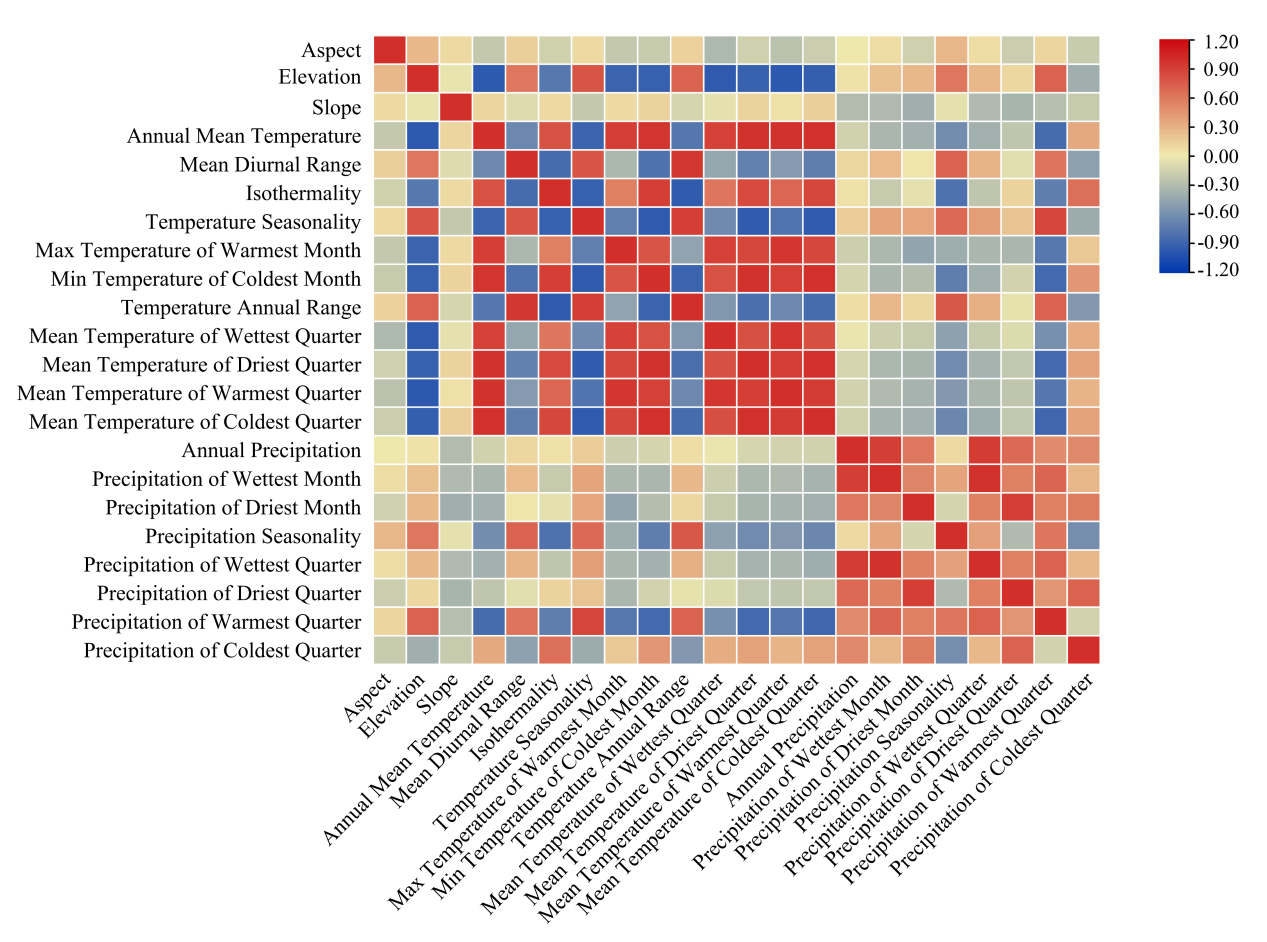
**

Supplement: Supplementary file 2 — Data S2: ece373793‐sup‐0002‐Supinfo02.docx. [file ECE3-16-e73793-s002.docx]
