# Supplementary material for: A Multi‐Factor Habitat Suitability Model for Asian Elephants in the Greater Mekong Subregion: Effects of Vegetation and Climate
Source: Ecol Evol. 2026 Jun 16;16(6):e73793. doi: 10.1002/ece3.73793 (PMC13270213; doi:10.1002/ece3.73793)
Supplement: Supplementary file 3 — Data S3: ece373793‐sup‐0003‐Supinfo03.docx. [file ECE3-16-e73793-s001.docx]

| 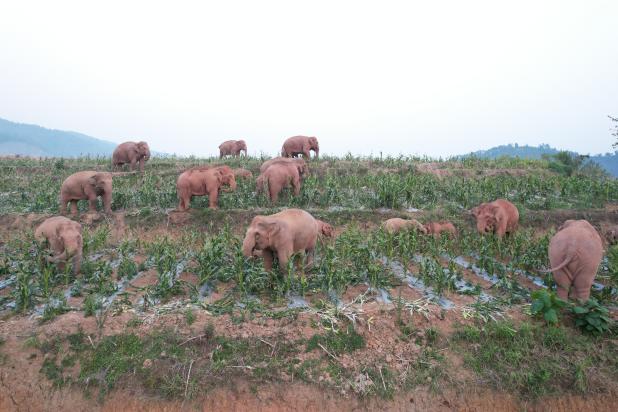 | 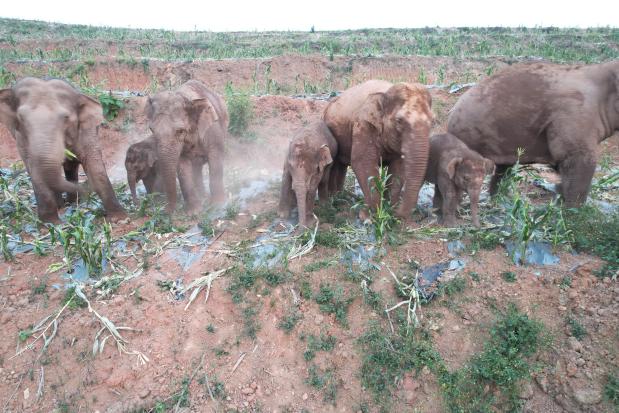 |
| --- | --- |
| 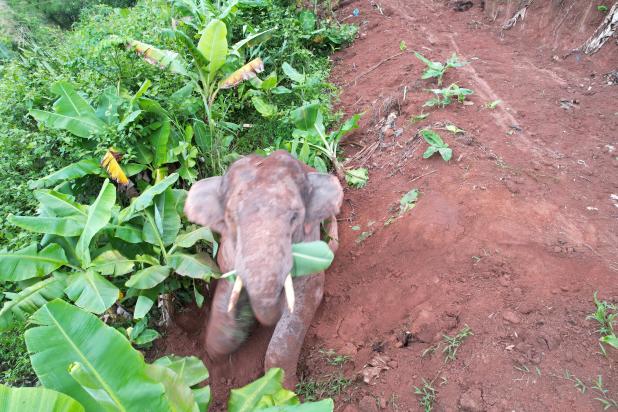 | 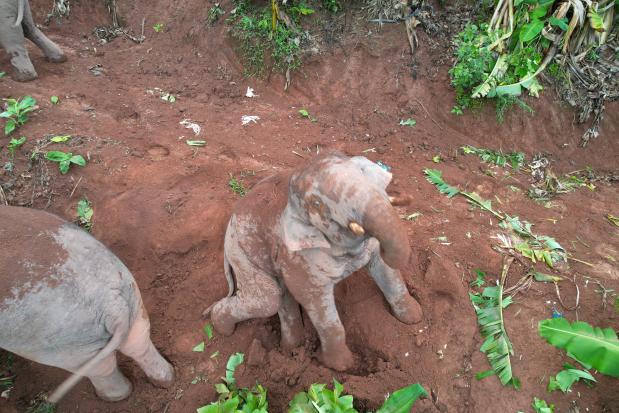 |
| 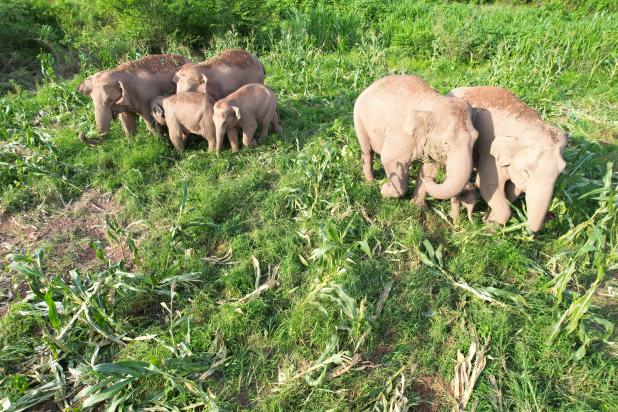 | 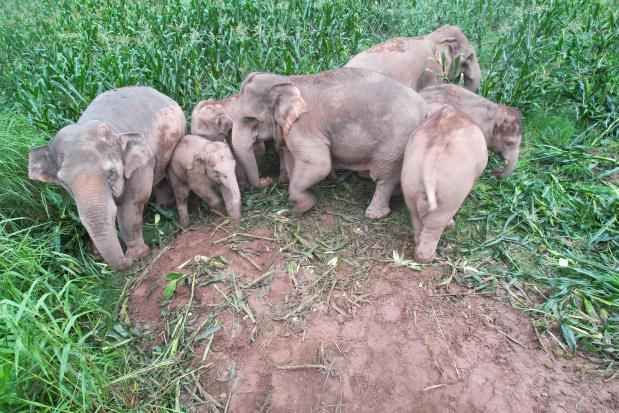 |

An Asian elephant engaging in crop-foraging behavior on agricultural land. (Provided by the Jiangcheng County Forestry and Grassland Bureau.)
